# The obesity epidemic – Nature via nurture: A narrative review of high-income countries

**DOI:** 10.1177/2050312120918265

**Published:** 2020-04-28

**Authors:** Sarah E Jackson, Clare H Llewellyn, Lee Smith

**Affiliations:** 1 Department of Behavioural Science and Health, University College London, London, UK; 2 Cambridge Centre for Sport and Exercise Sciences, Anglia Ruskin University, Cambridge, UK

**Keywords:** Epidemiology, public health, obesity, epidemic, gene–environment interaction, genetic influences, environmental influences, obesogenic environment

## Abstract

Over the last three decades, the prevalence of obesity has increased rapidly in populations around the world. Despite a wealth of research, the relative contributions of the different mechanisms underlying this global epidemic are not fully understood. While there is growing consensus that the rapid rise in obesity prevalence has been driven by changes to the environment, it is evident that biology plays a central role in determining who develops obesity and who remains lean in the current obesogenic environment. This review summarises evidence on the extent to which genes and the environment influence energy intake and energy expenditure, and as a result, contribute to the ongoing global obesity epidemic. The concept of genetic susceptibility to the environment driving human variation in body weight is discussed.

## Introduction: the obesity epidemic

Almost 20 years ago, the World Health Organization (WHO) declared the problem of rising levels of obesity a ‘global epidemic’,^1^ yet the prevalence of overweight (body mass index (BMI; a ratio of weight to height commonly used to categorise weight status) ⩾ 25 kg/m^2^) and obesity (BMI ⩾ 30 kg/m^2^) has continued to rise.^2,3^ In 2016, more than 1.9 billion adults (39% of the world’s adult population) were affected by overweight, of whom over 650 million (13%) had obesity,^4^ with obesity rates surpassing 50% in many countries.^3^ A high number of children and adolescents are also affected: in 2016, some 41 million children under the age of 5 years and 340 million (18%) of those aged 5–19 years had developed overweight or obesity.^4^ While the increase in obesity prevalence in developed countries appears to have slowed down over recent years, developing countries are catching up rapidly and no country has been successful in reversing the epidemic.^3^ Modelled estimates have forecasted a further 33% increase in obesity prevalence by 2030.^5^

What’s more, the distribution of BMI has shifted, so although people across the weight spectrum have become heavier, the change has been greatest at the upper end. In a landmark paper^6^ that compared data collected from US adults aged 20–74 years between 1976–1980 and 1999–2004 as part of the National Health and Nutrition Examination Survey (NHANES), it was clear to see not only that the distribution of BMI has shifted to the right over recent decades, but that the change has been greater at the upper centiles of the distribution. This indicates that the distribution has become more skewed. In addition to the twofold increase in the rate of obesity in the adult population (from 15.0% in 1976–1980 to 32.9% in 2003–2004), the proportion with a BMI in the range considered morbidly obese (BMI ⩾ 40 kg/m^2^) has more than tripled (from 1.4% in 1976–1980 to 5.1% in 2003–2004).^6^ Similar changes have also been observed in the United Kingdom^7^ and other high-income countries.^8,9^ Statistical forecasts suggest that the prevalence of morbid obesity will continue to increase rapidly, with a 130% rise in prevalence projected between 2010 and 2030.^5^ An increase in obesity is not just limited to westernised countries. For example, in 2015, China and India had the highest numbers of children with obesity globally and China, along with the United States, had the highest number of adults with obesity.^10^ The fastest growth rates in the prevalence of overweight and obesity have been observed in Africa and Asia.^11^

The recent increase in obesity prevalence has made understanding what causes obesity even more important. At the simplest level, a person can only develop obesity when their energy intake exceeds their energy expenditure over a long period, creating chronic positive energy balance. Two key factors that influence energy balance are food consumption (i.e. the energy density and quantity of food consumed) and physical activity (i.e. the type, intensity and frequency of activities carried out). Weight gain occurs when there is a greater consumption of energy (calories) than expenditure. Understanding the extent to which genes and the environment influence these factors, and as a result, contribute to the ongoing global obesity epidemic, is important for informing intervention, policy and practice. This review provides a summary of the current evidence, weighing up the relative importance of nurture and nature in the development of obesity. Given the breadth of the literature and differences in environments across high-, middle- and low-income countries, we focus here on the predominance of literature from high-income countries. For more information on middle- and low-income countries we recommend the review by Ford et al.^12^

## Nurture: the contribution of environmental influences on obesity

The obesity epidemic is often attributed to the ‘obesogenic’ modern environment, which imposes a wide range of barriers to maintaining a healthy weight.^13,14^ A growing body of evidence highlights the substantial influence of environmental factors on energy intake and energy expenditure that promote positive energy balance.

### Energy intake and obesity

Energy intake (the calories from fat, carbohydrate, protein and alcohol in food and drink consumed) has increased significantly in adults^15–18^ in developed countries worldwide since the 1970s. Evidence on changes in energy intake in children and adolescents is less consistent.^16,19–22^ A key driver is the changing environment.

In the developed world, there is easy access to large quantities of low-cost, high-calorie, high-fat foods. Recent decades have seen a dramatic change in the foodscape, with developments in food production, processing, storage and preparation making highly palatable and energy-dense foods cheaper and more accessible.^23^ The nutritional transition to processed foods and high-calorie diets^24^ has been driven in large part by an increase in the total number of food outlets, in particular those providing fast food. For example, a study in the United Kingdom showed the total number of food outlets increased by ~80% between 1980 and 2000.^25^ Importantly, exposure to takeaway food outlets in home, work and commuting environments combined has been found to be associated with greater consumption of takeaway food, higher BMI and increased risk of obesity.^26^

In addition to increases in the availability of processed and high-calorie foods, there has been a dramatic increase in portion sizes.^27–29^ Because many people find it difficult to regulate their food intake,^30^ this can have a considerable impact on energy intake. Experimental studies have shown that people consume more when they are offered larger portions; a finding consistently observed across meals,^31–33^ snacks^34^ and non-alcoholic beverages,^35^ and in adults^35,36^ and children.^35,37^ A meta-analytic review concluded that doubling the portion size increases consumption by 35%.^36^

Increased exposure to food cues may also contribute to the rise in energy intake. Food advertising is prominent in modern society, with a strong focus on less healthy foods. One study carried out in the United States^38^ found that food advertising now accounts for nearly half of all commercial messages on children’s programmes. An average hour included 11. The majority of foods adverts directed to children are for high-calorie, low-nutrient food products that should not be part of a regular diet.^38^ Exposure to unhealthy snack foods, such as at supermarket checkouts^39^ or in vending machines,^40^ may also serve to increase energy intake.

Socioeconomic disadvantage is also an important influence on energy intake and obesity. In developed countries, there is a well-established socioeconomic gradient in obesity, with the highest prevalence seen in groups with the lowest levels of education and income and in the most deprived areas.^41–44^ This appears to be particularly pronounced in childhood.^44–47^ What’s more, while overall trends for increasing prevalence of overweight and obesity have slowed or levelled off in many countries since the turn of the century, they have continued to rise among children and adolescents with greater social disadvantage, exacerbating socioeconomic disparities.^45,46,48^ Data show that individuals from lower socioeconomic groups tend to be less likely than those from middle and higher socioeconomic groups to have a healthy diet.^42,49^ The food environment likely plays a role, with affordable healthy foods less accessible but unhealthy convenience foods readily available in more deprived communities.^50,51^

On a global level, technological advances and changes in the regulatory environment have seen food systems in low- and middle-income countries change substantially over the past few decades. Globalised distribution of technology related to food production, transportation and marketing have seen traditional food markets replaced by large supermarkets which offer increased access to cheaper, processed foods that are high in fat, sugar and salt.^52^ World Trade Organization regulation has reduced barriers to food trade, allowing greater access to global commodities. These changes have led to diets in low- and middle-income countries becoming increasingly westernised, with higher intakes of refined carbohydrates, added sugars, fats and animal-source foods, and lower intakes of legumes, vegetables and grains.^52^ These changes to the food environment have been hypothesised to explain the rapid increases in both obesity and metabolic diseases observed in countries in transition.

Studies reporting substantial increases in body weight in people who are genetically prone to obesity who migrate from a less to more obesogenic environment clearly demonstrate the impact that a changing food environment can have on obesity risk.^53,54^ Tackling these changes in the food environment to help people more effectively manage their energy intake presents an ongoing challenge. Evidence is accumulating on strategies that may be put in place to reduce energy consumption,^55^ such as better food labelling^15^ and limiting the proximity of fast food restaurants to schools and workplaces.^56^ In the United States, some progress has been made in ‘detoxifying’ the environment, for example, by introducing calorie/nutrition labelling on menus, regulating food ingredients (e.g. trans fats), and in some regions, restricting food advertising and introducing a tax on sugar-sweetened beverages.^55^ In the United Kingdom, a childhood obesity plan aims to remove foods high in sugar, fat and salt from the checkouts of supermarkets and impose a ban on advertising of these foods on TV before 9 p.m.^57^

Measures such as these may help to reduce the risk of obesity both in the current population and future generations. There is evidence to suggest that even the food environment a person is exposed to before birth may influence energy intake and, as a result, risk of obesity later in life. Both under- and over-nutrition in utero appear to produce permanent alterations in neural circuits that control appetite, in particular, relating to leptin (a hormone linked to satiety).^58,59^ Animal models suggest that infants born to mothers who experience malnutrition during pregnancy are born with lower levels of leptin, resulting in a more avid appetite – potentially with the purpose of promoting ‘catch-up’ growth.^58,59^ This, when combined with an obesogenic environment, can increase risk of obesity. For infants who are overnourished in utero and born large, their hypothalamus is resistant to high levels of circulating leptin which reduces their satiety sensitivity and may also lead to obesity.^58,59^

### Energy expenditure and obesity

Energy expenditure is the sum of the basal metabolic rate (the amount of energy expended while at complete rest), the thermic effect of food (the energy required to digest and absorb food) and the energy expended in physical activity. The environment is an important influence on the latter component of this equation by facilitating or limiting opportunity for physical activity.

Limited literature exists on secular trends in physical activity over recent decades, because reliable measures to monitor population levels of free-living physical activity have only recently been introduced.^60^ However, what literature does exist suggests that there has been a reduction in several domains of physical activity. For example, a large-scale study of adults in Finland observed a decline in occupational physical activity, with the proportion of men and women in physically demanding work decreasing from 60% to 38% and 47% to 25%, respectively, between 1972 and 2002.^61^ Daily commuting physical activity also decreased over the same time period, from 30% to 10% in men and from 34% to 22% in women.^61^ However, reductions were not universally observed across all domains of physical activity; there was an increase in the proportion engaging in leisure-time physical activity, from 66% to 77% in men and from 49% to 76% in women.^61^ Data from US surveys have shown similar trends, with substantial reductions in occupational, transportation and home-based physical activity and an increase in sedentary time contributing to an overall reduction in total physical activity, despite a small increase in leisure-time physical activity.^62^ While limited data exist on secular trends in physical activity, prevalence data are available. In 2016, the lowest levels of physical activity were in men from Oceania (12.3%), East and Southeast Asia (17.6%), and sub-Saharan Africa (17.9%).^63^ Prevalence of physical activity in 2016 was more than twice as high in high-income countries (36.8%) as in low-income countries (16.2%).^63^ Such data provide further support that obesity is a global epidemic.

A key contributor to the decline in physical activity has been the development of new technologies that have facilitated the automation of industry and increased leisure-time sedentary behaviour. In the early 1960s, almost half of jobs in private industry in the United States required at least moderate-intensity physical activity; today it is less than one in five.^64^ As a result, the estimated mean daily energy expenditure attributable to work-related physical activity has fallen by more than 100 calories.^64^ Given that people of working age typically spend the majority of their waking hours at work, the decline in occupational energy expenditure is unlikely to be offset by the small increase in leisure-time physical activity that has been observed.^61,62^

The recent and widespread surge in the availability of screen-based leisure activities (e.g. television, computer, video games) has also contributed to obesity by encouraging greater sedentary leisure time.^65,66^ It is important to note that sedentary behaviour is not simply the absence of physical activity, and in fact does not appear to displace time in physical activity.^67^ Rather, sedentary behaviour encompasses activities where sitting or reclining is the dominant mode of posture and energy expenditure is very low. Accumulating evidence supports a causal relationship between time spent in sedentary activities and the development of obesity, at least in childhood and adolescence.^66–68^

Another aspect of the changing environment thought to contribute to obesity through a reduction of physical activity is urban sprawl. This is the expansion of human populations away from central urban areas into low-density, monofunctional and usually car-dependent communities. In other words, it is the spread of an urban area into what used to be countryside. The rate of urban sprawl has increased in recent decades,^69^ likely owing to an increasing global population. Importantly, urban sprawl has been shown to be associated with negative health outcomes for those residing in such areas including an increase in overweight and obesity.^70,71^ This may be a consequence of reduced physical activity for those residing in such areas, for example, resulting from a dependency on motorised transport to access common destinations (e.g. work, school, shops).^70^ Multi-country studies have shown that low- and middle-income countries with greater levels of urbanisation and economic development tend to have lower levels of physical activity.^72,73^

Linked to urban sprawl, there has also been a substantial increase in car use. For example, in the United Kingdom, there are over 2.5 million new car registrations annually, with an estimated 470 cars per 1000 people.^74^ The proportion of households with at least one car increased from 14% in 1951 to 78% in 2008.^74^ Car use increased from an average of 429 trips per person in 1976 to 613 in 2009.^74^ Replacing car travel with more active modes (e.g. walking/cycling) could significantly improve physical activity rates and have a resultant impact on overweight and obesity.^75^ It should be noted that increasing car ownership and use is not just limited to Westernised countries. For example, in China, car ownership per capita has grown in the 2000s at a compound rate of approximately 21% per annum.^76^ Moreover, there are currently more new cars being sold annually in China (21.1 million) than in either the European Union (14.3 million) or North America (9.2 million).^77^

Overall, there is agreement that net physical activity is less than it was 50 years ago, and that this contributes to positive energy imbalance and the development of obesity. Evidence points to clear health benefits of physical activity for people with overweight and obesity, even in the absence of clinically significant weight loss.^78^ For a detailed review of the literature on physical activity and health, see Fletcher et al.^79^ Research is now exploring aspects of the physical environment that could be manipulated to increase population levels of physical activity. For example, greater accessibility to common destinations (e.g. parks, shops), new infrastructure for active travel (walking, cycling) and public transport, and land use mix have all been shown to encourage higher levels of physical activity.^80^

Considering the range of forces acting to reduce physical activity and increase energy intake, it is little wonder that a large proportion of the population has developed overweight and obesity. Indeed, James^81^ described the obesity epidemic as a ‘normal population response to the dramatic reduction in the demand for physical activity and the major changes in the food supply of countries over the last 40 years’.

## Nature: the contribution of genetic influences on obesity

The obesogenic environment goes some way towards explaining the rapid increase in obesity prevalence over recent decades, but environmental factors are less good at explaining why some people develop obesity while others maintain a moderate body weight with relative ease, when exposed to similar environments. Genetic factors are hypothesised to explain a large proportion of the variation in susceptibility to obesity. The concept of genetic contribution to obesity has long been acknowledged, with evidence of the tendency towards obesity to vary between families reported as early as 1923.^82^ Over recent decades, the extent of genetic influence on BMI has been estimated using twin studies, which compare monozygotic (MZ, identical) twins, who share 100% of their genes, with dizygotic (DZ, non-identical) twins, who share on average 50% of their segregating genes. Greater similarity in BMI between the MZ twin pairs compared with the DZ twin pairs points to a genetic contribution to BMI. The proportion of variation in BMI is quantified with a ‘heritability’ statistic, which ranges from 0% (indicating genetic variation plays no role in explaining the variability in BMI) to 100% (indicating genetic variation entirely explains the variability in BMI). Using this method, twin studies have provided a wealth of evidence for high heritability of BMI across the life course. A large meta-analysis of 31 twin studies (n = 140,525) showed that for adults, heritability estimates of BMI range from 47% to 90%.^83^ Similarly, another meta-analysis of 45 twin studies conducted in children (n = 175,564) observed consistently high heritability estimates for BMI across childhood and adolescence (range: 41%–85%), with heritability increasing from mid-childhood (~42%) to the onset of adulthood (75%).^84^ These results indicate that genetic factors play a major role in the variation of BMI among populations of different ethnicities exposed to different environmental factors related to obesity.

Identifying the genes that explain this variation provides an ongoing challenge. In 2007, a breakthrough was made by Frayling et al.,^85^ who reported consistent associations between variants of the fat mass and obesity-associated gene (FTO) and adiposity in adults and children. Compared with those who carry two copies of the low-risk variant, adults who carry one copy of the high-risk variant are on average 1.2 kg heavier, and those who carry two copies of the high-risk variant are on average 3 kg heavier. The discovery of FTO was an important advance in understanding the genetic drivers of obesity for two reasons: (1) around half of the population carries at least one of the high-risk variants, and (2) the effect size was large enough for researchers to explore its mechanisms. Since the link between body weight and FTO was identified, genome-wide meta-analyses have identified close to 1000 single nucleotide polymorphisms that are reliably associated with BMI.^86^ Nevertheless, these variants are only able to account for 6% of the variance in BMI; a tiny proportion of the amount thought to be explained by genes.^86^ The remaining genetic variance is thought to be due to rare genes, a very large number of common genes of very small effect, and gene–gene and gene–environment interactions.

## Nature *and* nurture: interplay between genes and the environment

A key question that researchers have been trying to answer is *how* genes confer differential obesity risk in the context of the modern obesogenic environment. There is convincing evidence that, in fact, obesity develops from a complex interaction between genetic susceptibility and exposure to a obesogenic environment,^87^ or as Bray^88^ famously stated, ‘Genes load the gun, the environment pulls the trigger’. Such an interaction is suggested by the trends in obesity prevalence. While absolute numbers of individuals with overweight and obesity have risen notably over recent years (evidence of the increasingly obesogenic environment), the distribution of BMI has become increasingly skewed such that the proportion of individuals at the heavier end of the spectrum – that is, those with severe obesity – has risen disproportionately.^6,7,89^ This suggests that people at higher genetic risk of obesity are particularly susceptible to the modern obesogenic environment.

**Figure 1.**
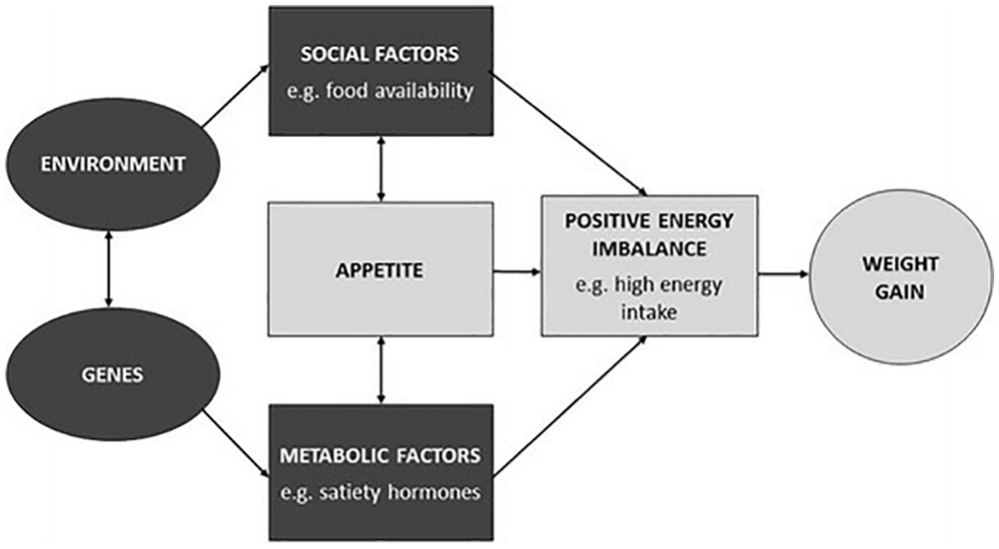
Behavioural susceptibility theory: how appetite mediates the interaction between genetic susceptibility to obesity and environmental exposure. Individuals who inherit a set of genes that bestow greater responsiveness to external food cues and/or lower sensitivity to satiety are more likely to overeat in response to an ‘obesogenic’ food environment, and to gain excessive weight. Obesity, therefore, results from a combination of genetic susceptibility to overeating and exposure to an ‘obesogenic’ food environment. Source: Reproduced from Llewellyn and Fildes^91^ with permission from the authors.

The basis of this gene–environment interaction has received a great deal of attention in recent years. One explanatory theory with a growing evidence base is that of behavioural susceptibility for obesity.^90^ Proposed in 2007 by the late Professor Jane Wardle, behavioural susceptibility theory (BST) of obesity hypothesises that genetic susceptibility to obesity operates via appetitive mechanisms. The key idea is that individuals who inherit a more avid appetite are more likely to overeat in response to the opportunities offered by the current food environment and to develop obesity – that is, obesity results partly from genetic susceptibility to overeating in the context of an obesogenic environment (Figure 1).^90,92^ The appetitive traits of food responsiveness (i.e. a person’s tendency to want to eat in response to the sight, smell or taste of palatable food) and satiety responsiveness (the extent to which a person eats when they are hungry and stops when they are full) are thought to be of particular importance. Individuals who are genetically predisposed to be highly responsive to food cues are more likely to overeat in an environment in which highly palatable food is promoted aggressively and readily available. Those predisposed to weaker satiety signals are more likely to overeat in response to larger portion sizes and multiple opportunities to eat.

BST has gained traction because it is supported by several study designs. Many of the BMI-associated common genetic variants are located in or near genes that are involved in the central control of energy balance and, in particular, appetite regulation.^93^ For example, gene expression studies have shown enrichment in the hypothalamus, pituitary gland (both are key structures involved in the regulation of hunger and satiety), hippocampus and limbic system, suggesting that a range of neuropsychological processes that influence eating behaviour may be involved, such as emotion, cognition, learning and memory.^93^

Large population-based studies have also demonstrated sub-stantial variation in appetite which is (1) moderately to highly heritable during infancy,^94^ childhood^95,96^ and adulthood;^97–99^ and (2) both associated with measured genetic risk of obesity in childhood^100^ and adulthood,^101–104^ and mediates part of the genes-BMI association. Variation in appetite also predicts rates of prospective weight gain from infancy to toddlerhood.^105–107^ Findings in children and adults have been mixed, and suggest a more complex bidirectional relationship that is moderated by other psychosocial factors such as cognitive restraint over eating and depression.^108–112^ Taken together, these studies suggest that there are large individual differences in appetite regulation that have a genetic basis, and have the potential to influence weight gain through exposure to an environ-ment with increased opportunity to eat – that is, an ‘obesogenic’ environment.

In line with this, a wealth of studies have shown that genetic influence on weight is stronger in samples exposed to a more ‘obesogenic’ environment, indexed at the macro level, as well as the level of the community/home environment, and on an individual basis. In particular, twin study estimates of the heritability of BMI are higher in samples drawn from countries with a higher gross domestic product (GDP),^113^ in populations with a higher average BMI,^113^ in families of lower socioeconomic status and educational attainment,^114–116^ and in samples with a later year-of-birth who have spent a greater proportion of their lifespan living in an ‘obesogenic’ environment.^117^ In keeping with twin findings, molecular genetic studies have also reported stronger associations between measured genetic risk of obesity and BMI in cohorts with a more recent year of birth,^118^ and among children and adults of lower socioeconomic status.^119,120^ In a large study of 120,000 older adults from the UK Biobank, carrying an additional 10 BMI-increasing genetic variants was associated with less increased weight for those in the top versus the bottom 50% of socioeconomic position (2.9 vs 3.8 kg).^120^ A rather intriguing observation is the large increase in the heritability of BMI from early childhood (41%) to the onset of adulthood (75%), and a corresponding diminishing of the shared family environmental influence, from an analysis of 45 twin cohorts from 20 countries (87,782 twin pairs; nearly 400,000 BMI measurements).^84^ Associations between BMI and measured genetic risk also strengthen after early childhood,^121–126^ suggesting, somewhat paradoxically, that as individuals gain increasing exposure to the obesogenic environment, genetic influence on weight increases. However, inherent in BST is the idea that ‘obesity genes’ cannot be fully expressed unless an individual has the freedom and opportunity to consume as much and as often as they wish; a privilege that develops with age. The increasing age-related heritability of BMI during childhood may therefore reflect children’s growing autonomy to act in line with their genetically influenced appetitive traits. This phenomenon has been termed ‘gene–environment correlation’ – the notion that individuals actively interact with their environment in ways that reflect their genetic predispositions.

At the more proximal community or home environment level, a large twin study showed that the heritability of BMI was substantially lower among children living in a ‘healthy’ versus ‘obesogenic’ environment (39% vs 86%), characterised by the structural and social aspects of the home food, media and physical activity environments.^127^ Among adults, heritability of BMI was lower among those living in an environment that provided greater opportunity for physical activity (‘walkability’).^128^

At the individual micro-level, undertaking more physical activity mitigates both the genetic propensity towards a higher BMI^128–130^ and weight gain,^131^ as estimated from twin studies. Numerous studies of measured genetic risk have converged with these twin findings, reporting a more pronounced association between BMI-associated genetic variants and BMI among physically inactive individuals.^132,133^ In particular, a large-scale meta-analysis of 218,166 adults showed that being physically active attenuates the BMI-increasing effect of variants in the FTO gene by ~30%.^134^ The large UK Biobank study found that a composite score of the individual-level ‘obesogenic’ environment (which included physical activity, sedentary behaviour, time spent watching television, and Westernised diet) moderated genetic predisposition to be of a higher or lower BMI.^120^ When components were examined individually, higher levels of physical activity and fewer hours spent watching television were the behaviours that partly offset genetic predisposition to higher BMI.^120^ The protection conferred by physical activity from genetic susceptibility to obesity may partly reflect the role that habitual physical activity is thought to play in optimising appetite regulation (by upregulating satiety sensitivity).^135,136^

## Limitations

This review summarises the literature on genetic and environmental influences on body weight. Given the broad scope, a narrative approach was favoured over a systematic literature search. While this has benefits in permitting a breadth of evidence to be included, the absence of a systematic search strategy introduces potential for biased selection of studies. There is a substantial literature on this topic and we recommend reading widely to gain a fuller picture of the different approaches that have been taken to study the causes of the obesity epidemic and the various strategies that have been proposed (and undertaken) in an effort to curtail the problem.^55,78,137^

## Conclusion

The aetiology of obesity is complex and multifactorial. While there is growing consensus that the rapid rise in obesity prevalence has been driven by changes to the environment, it is evident that biology plays a central role in determining who develops obesity and who remains lean. In particular, genetically predetermined appetitive traits may have an important influence on the extent to which the current ‘obesogenic’ environment maximises genetic expression of body weight. The interaction between genetic susceptibility to obesity and the ‘obesogenic’ environment is a growing area of research that is starting to yield important insights for public health initiatives. Obesity is not simply a lifestyle choice; rather it results from a complex interaction between genetic susceptibility and exposure to an environment that encourages positive energy balance. The individuals who are at greatest risk are those who are both at high genetic susceptibility and are living in an environment that makes ‘healthy choices’ more difficult, such as those living in deprived areas. Thus, as is typically the case in debates of genetic versus environmental contribution to any phenotype, when it comes to the obesity epidemic, it is not nature or nurture; rather, it is nature *via* nurture.

The gene–environment interplay in the aetiology of obesity has implications for public health policy and clinical practice. In terms of policy, the evidence base overwhelmingly suggests that greater regulation of the wider food environment and creating more opportunity for physical activity would offset obesity risk both for individuals at high genetic susceptibility to obesity and for those living in deprivation. There is a need for further research into initiatives that can bring about meaningful change in the food and physical activity environments that will protect individuals who are at high genetic susceptibility to environmental pressures. In terms of practice, clinicians should be provided with training to assess and effectively counsel patients on lifestyle factors that contribute to obesity. However, they should be advised to maintain an awareness that body weight is influenced by a broad range of factors, many of which are outside of personal control, and bear this in mind when recommending weight loss to patients. The simple ‘eat less and move more’ mantra is unhelpful and does not take into account that weight-related behaviours are highly context-dependent and are influenced by a large number of biopsychosocial factors.^138^
